# Supplementary material for: The Impact of New Surgical Techniques on Geographical Unwarranted Variation: The Case of Benign Hysterectomy
Source: Int J Environ Res Public Health. 2021 Jun 22;18(13):6722. doi: 10.3390/ijerph18136722 (PMC8297008; doi:10.3390/ijerph18136722)

Supplementary materials

Appendix 1: ICDM-9CM codes used to select patients who underwent benign hysterectomy and codes for post-operative complications

The two tables report the ICDM-9CM international codes to identify procedures and diagnosis, as well as to define the exclusion criteria of patients. They allow the selection of the same patients in order to ease replicability

Table S1 Codes for Benign Hysterectomy patient identification

**Minimally invasive**<br>(ICD-9-CM Procedures codes in the hospital admission data in in all positions – from principal to sixth procedures):<br>**68.31; 68.41**
**Open**<br>(ICD-9-CM Procedures codes in the hospital admission data in in all positions – from principal to sixth procedures):<br>**68.39; 68.49; 68.9**
**Exclusions**<br>(ICD-9-CM Diagnosis codes in the hospital admission data in first position):<br>179*; 180*; 181*; 182*; 183*; 184*; 869*; 2331; 2332; 2333; 236.0; 236.1; 236.2; 236.3; 867.4; 867.5; 867.6; 867.7; 867.8; 867.9; 868.00; 868.03; 868.04; 868.09; 868.10; 868.13; 868.14; 868.19; 879.6; 879.7; 867.9; 879.8; 879.9; 906.0; 908.1; 908.2; 939.1; 947.4; 233.30; 233.31; 233.32;233.39
(Department codes):<br>28 (Spinal Unit); 56 (Functional Rehabilitation); 60 (Long-term care) ; 75 (Neuro Rehabilitation)
MDC codes<br>14 (Pregnancy, Childbirth & the Puerperium)

Table S2 Codes for post-operative complications

ICD-9-CM Procedures codes in the hospital admission data in in all positions – from principal to sixth procedures:<br>54.12; 54.61; 86.59; 54.21; 54.11; 54.19; 39.79; 70.71; 96.14; 71.22; 70.12; 97.83; 46.03; 46.11; 45.76; 45.62; 4591; 4621; 5359; 5732; 5781; 598
ICD-9-CM Diagnosis codes in the hospital admission data in first position:<br>998.11; 998.12 285.1 568.81 998.59 593.3 596.1 593.82 619.0 619.1 591 596.6 599.7 620.7 990.3 553.1 553.20 553.21 560.9 560.1 560.2 567.21 614.4

Appendix 2: ALOS by provider group

The three graphs display the ALOS by provider, divided by provider group (A or B) for all the procedures, only open and only minimally-invasive ones.

Figure S6: Average length of stay (ALOS) related to all procedures (open and minimally-invasive procedures)

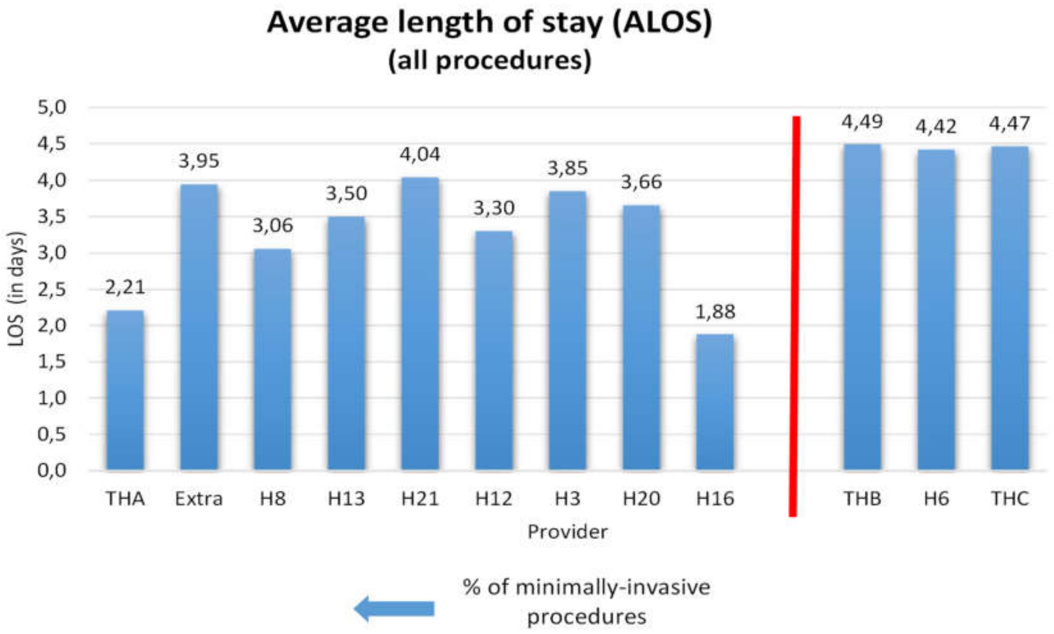

Figure S7: Average length of stay (ALOS) of procedures performed with minimally-invasive technique

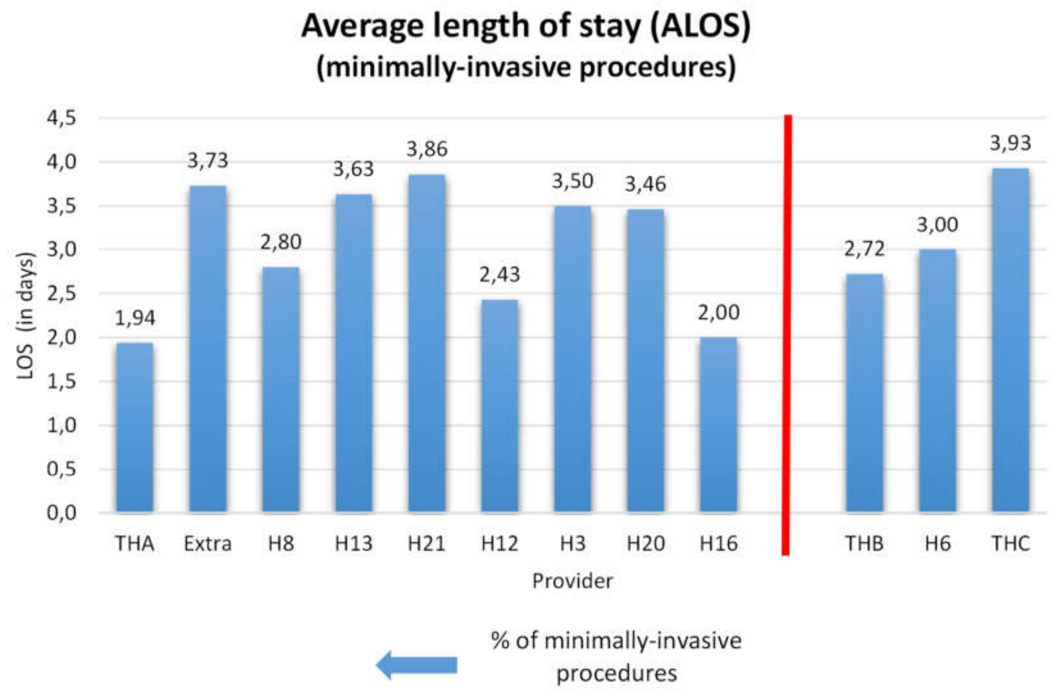

Figure S8: Average length of stay (ALOS) of procedures performed with open technique

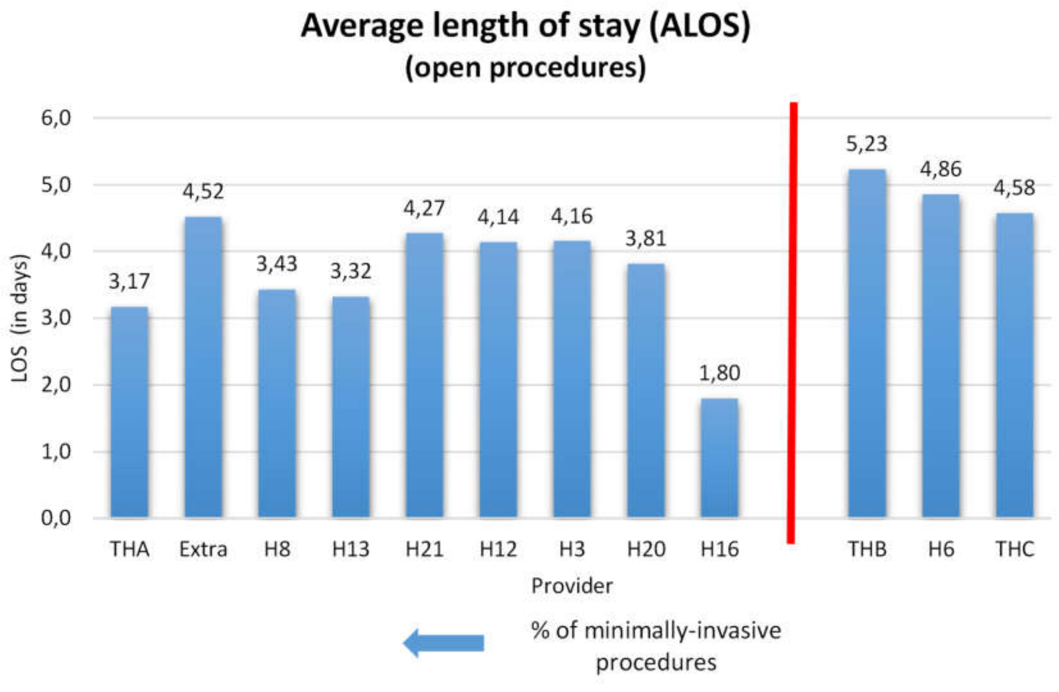

Appendix 3 Complication rates of Tuscan providers combined with respective percentages of procedures performed with minimally invasive technique

The graph displays post-operative complication rates at a provider level, and highlights the differences between providers belonging to group A and B.

Figure S9: Complication rates of Tuscan providers combined with respective percentages of procedures performed with minimally invasive technique

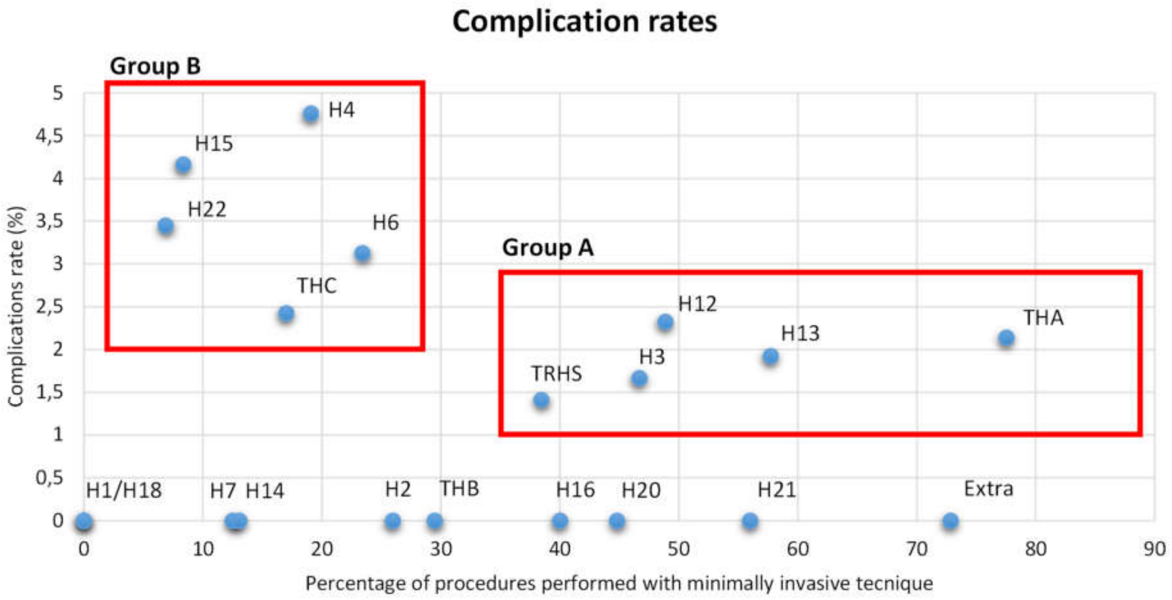

Supplement: Supplementary file 1 [file ijerph-18-06722-s001.zip › ijerph-1258791-supplementary.pdf]
